# Supplementary material for: A fault-intrusion-tolerant system and deadline-aware algorithm for scheduling scientific workflow in the cloud
Source: PeerJ Comput Sci. 2021 Nov 2;7:e747. doi: 10.7717/peerj-cs.747 (PMC8576554; doi:10.7717/peerj-cs.747)
Supplement: Supplemental Information 6 [file peerj-cs-07-747-s006.zip › Myproject1.1/docs/index-files/index-3.html]

C-Index


JavaScript is disabled on your browser.


- Overview
- Package
- Class
- Use
- Tree
- Deprecated
- Index
- Help

- Prev Letter
- Next Letter

- Frames
- No Frames

- All Classes

A B C D E F G H I J L M N O P Q R S T U V W Z 


## C

cachedcloudlet - Static variable in class org.cloudbus.cloudsim.network.datacenter.NetDatacenterBroker


cancel(int, Predicate) - Static method in class org.cloudbus.cloudsim.core.CloudSim
:   Removes an event from the event queue.

cancelAll(int, Predicate) - Static method in class org.cloudbus.cloudsim.core.CloudSim
:   Removes all events that match a given predicate from the future event queue returns true if
    at least one event has been cancelled; false otherwise.

CANCELED - Static variable in class org.cloudbus.cloudsim.Cloudlet
:   The Cloudlet has been canceled.

cancelEvent(Predicate) - Method in class org.cloudbus.cloudsim.core.SimEntity
:   Cancel the first event matching a predicate waiting in the entity's future queue.

checkCloudletCompletion() - Method in class org.cloudbus.cloudsim.Datacenter
:   Verifies if some cloudlet inside this PowerDatacenter already finished.

checkCloudletCompletion() - Method in class org.workflowsim.DatacenterExtended
:   Verifies if some cloudlet inside this PowerDatacenter already finished.

ChildAwareHorizontalClustering - Class in org.workflowsim.clustering.balancing.methods
:   ChildAwareHorizontalClsutering is a method that merges a task and its children

ChildAwareHorizontalClustering(Map, Map, int) - Constructor for class org.workflowsim.clustering.balancing.methods.ChildAwareHorizontalClustering
:   Initialize a ChildAwareHorizontalClustering object

clean() - Method in class org.workflowsim.clustering.BasicClustering
:   Delete the root task

cleanTaskSetChecked() - Method in class org.workflowsim.clustering.balancing.BalancedClustering
:   Clean the checked flag of a taskset

cleanTaskSetChecked() - Method in class org.workflowsim.clustering.balancing.methods.BalancingMethod
:   Clean all the check points

clear() - Method in class org.cloudbus.cloudsim.core.DeferredQueue
:   Clears the queue.

clear() - Method in class org.cloudbus.cloudsim.core.FutureQueue
:   Clears the queue.

clearDatacenters() - Method in class org.cloudbus.cloudsim.DatacenterBroker
:   Destroy the virtual machines running in datacenters.

clearDatacenters() - Method in class org.cloudbus.cloudsim.network.datacenter.NetDatacenterBroker
:   Submit cloudlets to the created VMs.

clist - Variable in class org.cloudbus.cloudsim.network.datacenter.AppCloudlet


clock() - Static method in class org.cloudbus.cloudsim.core.CloudSim
:   Get the current simulation time.

clone() - Method in class org.cloudbus.cloudsim.core.SimEntity
:   Get a clone of the entity.

clone() - Method in class org.cloudbus.cloudsim.core.SimEvent
:   Create an exact copy of this event.

CloudInformationService - Class in org.cloudbus.cloudsim.core
:   A Cloud Information Service (CIS) is an entity that provides cloud resource registration,
    indexing and discovery services.

CloudInformationService(String) - Constructor for class org.cloudbus.cloudsim.core.CloudInformationService
:   Allocates a new CloudInformationService object.

Cloudlet - Class in org.cloudbus.cloudsim
:   Cloudlet is an extension to the cloudlet.

Cloudlet(int, long, int, long, long, UtilizationModel, UtilizationModel, UtilizationModel) - Constructor for class org.cloudbus.cloudsim.Cloudlet
:   Allocates a new Cloudlet object.

Cloudlet(int, long, int, long, long, UtilizationModel, UtilizationModel, UtilizationModel, boolean, List<String>) - Constructor for class org.cloudbus.cloudsim.Cloudlet
:   Allocates a new Cloudlet object.

Cloudlet(int, long, int, long, long, UtilizationModel, UtilizationModel, UtilizationModel, List<String>) - Constructor for class org.cloudbus.cloudsim.Cloudlet
:   Allocates a new Cloudlet object.

Cloudlet(int, long, int, long, long, UtilizationModel, UtilizationModel, UtilizationModel, boolean) - Constructor for class org.cloudbus.cloudsim.Cloudlet
:   Allocates a new Cloudlet object.

CLOUDLET\_CANCEL - Static variable in class org.cloudbus.cloudsim.core.CloudSimTags
:   Cancels a Cloudlet submitted in the CloudResource entity.

CLOUDLET\_CHECK - Static variable in class org.workflowsim.WorkflowSimTags


CLOUDLET\_LENGTH - Static variable in class org.cloudbus.cloudsim.examples.power.Constants


CLOUDLET\_MOVE - Static variable in class org.cloudbus.cloudsim.core.CloudSimTags
:   Moves a Cloudlet to another CloudResource entity.

CLOUDLET\_MOVE\_ACK - Static variable in class org.cloudbus.cloudsim.core.CloudSimTags
:   Moves a Cloudlet to another CloudResource entity with an acknowledgement.

CLOUDLET\_PAUSE - Static variable in class org.cloudbus.cloudsim.core.CloudSimTags
:   Pauses a Cloudlet submitted in the CloudResource entity.

CLOUDLET\_PAUSE\_ACK - Static variable in class org.cloudbus.cloudsim.core.CloudSimTags
:   Pauses a Cloudlet submitted in the CloudResource entity with an acknowledgement.

CLOUDLET\_PES - Static variable in class org.cloudbus.cloudsim.examples.power.Constants


CLOUDLET\_RESUME - Static variable in class org.cloudbus.cloudsim.core.CloudSimTags
:   Resumes a Cloudlet submitted in the CloudResource entity.

CLOUDLET\_RESUME\_ACK - Static variable in class org.cloudbus.cloudsim.core.CloudSimTags
:   Resumes a Cloudlet submitted in the CloudResource entity with an acknowledgement.

CLOUDLET\_RETURN - Static variable in class org.cloudbus.cloudsim.core.CloudSimTags
:   Denotes the return of a Cloudlet back to sender.

CLOUDLET\_STATUS - Static variable in class org.cloudbus.cloudsim.core.CloudSimTags
:   Denotes the status of a Cloudlet.

CLOUDLET\_SUBMIT - Static variable in class org.cloudbus.cloudsim.core.CloudSimTags
:   Denotes the submission of a Cloudlet.

CLOUDLET\_SUBMIT\_ACK - Static variable in class org.cloudbus.cloudsim.core.CloudSimTags
:   Denotes the submission of a Cloudlet with an acknowledgement.

CLOUDLET\_UPDATE - Static variable in class org.workflowsim.WorkflowSimTags


CLOUDLET\_UTILIZATION\_SEED - Static variable in class org.cloudbus.cloudsim.examples.power.random.RandomConstants


cloudletCancel(int) - Method in class org.cloudbus.cloudsim.CloudletScheduler
:   Cancels execution of a cloudlet.

cloudletCancel(int) - Method in class org.cloudbus.cloudsim.CloudletSchedulerSpaceShared
:   Cancels execution of a cloudlet.

cloudletCancel(int) - Method in class org.cloudbus.cloudsim.CloudletSchedulerTimeShared
:   Cancels execution of a cloudlet.

cloudletCancel(int) - Method in class org.cloudbus.cloudsim.network.datacenter.NetworkCloudletSpaceSharedScheduler
:   Cancels execution of a cloudlet.

cloudletFinish(ResCloudlet) - Method in class org.cloudbus.cloudsim.CloudletScheduler
:   Processes a finished cloudlet.

cloudletFinish(ResCloudlet) - Method in class org.cloudbus.cloudsim.CloudletSchedulerDynamicWorkload
:   Processes a finished cloudlet.

cloudletFinish(ResCloudlet) - Method in class org.cloudbus.cloudsim.CloudletSchedulerSpaceShared
:   Processes a finished cloudlet.

cloudletFinish(ResCloudlet) - Method in class org.cloudbus.cloudsim.CloudletSchedulerTimeShared
:   Processes a finished cloudlet.

cloudletFinish(ResCloudlet) - Method in class org.cloudbus.cloudsim.network.datacenter.NetworkCloudletSpaceSharedScheduler
:   Processes a finished cloudlet.

cloudletList - Variable in class org.cloudbus.cloudsim.DatacenterBroker
:   The cloudlet list.

cloudletList - Static variable in class org.cloudbus.cloudsim.examples.power.RunnerAbstract
:   The cloudlet list.

CloudletList - Class in org.cloudbus.cloudsim.lists
:   CloudletList is a collection of operations on lists of Cloudlets.

CloudletList() - Constructor for class org.cloudbus.cloudsim.lists.CloudletList


cloudletlist - Variable in class org.cloudbus.cloudsim.network.datacenter.NetworkVm


cloudletPause(int) - Method in class org.cloudbus.cloudsim.CloudletScheduler
:   Pauses execution of a cloudlet.

cloudletPause(int) - Method in class org.cloudbus.cloudsim.CloudletSchedulerSpaceShared
:   Pauses execution of a cloudlet.

cloudletPause(int) - Method in class org.cloudbus.cloudsim.CloudletSchedulerTimeShared
:   Pauses execution of a cloudlet.

cloudletPause(int) - Method in class org.cloudbus.cloudsim.network.datacenter.NetworkCloudletSpaceSharedScheduler
:   Pauses execution of a cloudlet.

cloudletReceivedList - Variable in class org.cloudbus.cloudsim.DatacenterBroker
:   The cloudlet received list.

cloudletResume(int) - Method in class org.cloudbus.cloudsim.CloudletScheduler
:   Resumes execution of a paused cloudlet.

cloudletResume(int) - Method in class org.cloudbus.cloudsim.CloudletSchedulerSpaceShared
:   Resumes execution of a paused cloudlet.

cloudletResume(int) - Method in class org.cloudbus.cloudsim.CloudletSchedulerTimeShared
:   Resumes execution of a paused cloudlet.

cloudletResume(int) - Method in class org.cloudbus.cloudsim.network.datacenter.NetworkCloudletSpaceSharedScheduler
:   Resumes execution of a paused cloudlet.

CloudletScheduler - Class in org.cloudbus.cloudsim
:   CloudletScheduler is an abstract class that represents the policy of scheduling performed by a
    virtual machine.

CloudletScheduler() - Constructor for class org.cloudbus.cloudsim.CloudletScheduler
:   Creates a new CloudletScheduler object.

CloudletSchedulerDynamicWorkload - Class in org.cloudbus.cloudsim
:   CloudletSchedulerDynamicWorkload implements a policy of scheduling performed by a virtual machine
    assuming that there is just one cloudlet which is working as an online service.

CloudletSchedulerDynamicWorkload(double, int) - Constructor for class org.cloudbus.cloudsim.CloudletSchedulerDynamicWorkload
:   Instantiates a new vM scheduler time shared.

CloudletSchedulerSpaceShared - Class in org.cloudbus.cloudsim
:   CloudletSchedulerSpaceShared implements a policy of scheduling performed by a virtual machine.

CloudletSchedulerSpaceShared() - Constructor for class org.cloudbus.cloudsim.CloudletSchedulerSpaceShared
:   Creates a new CloudletSchedulerSpaceShared object.

CloudletSchedulerTimeShared - Class in org.cloudbus.cloudsim
:   CloudletSchedulerTimeShared implements a policy of scheduling performed by a virtual machine.

CloudletSchedulerTimeShared() - Constructor for class org.cloudbus.cloudsim.CloudletSchedulerTimeShared
:   Creates a new CloudletSchedulerTimeShared object.

cloudletsSubmitted - Variable in class org.cloudbus.cloudsim.DatacenterBroker
:   The cloudlets submitted.

cloudletsSubmitted - Variable in class org.workflowsim.ClusteringEngine
:   The number of tasks submitted.

cloudletSubmit(Cloudlet, double) - Method in class org.cloudbus.cloudsim.CloudletScheduler
:   Receives an cloudlet to be executed in the VM managed by this scheduler.

cloudletSubmit(Cloudlet) - Method in class org.cloudbus.cloudsim.CloudletScheduler
:   Receives an cloudlet to be executed in the VM managed by this scheduler.

cloudletSubmit(Cloudlet) - Method in class org.cloudbus.cloudsim.CloudletSchedulerDynamicWorkload
:   Receives an cloudlet to be executed in the VM managed by this scheduler.

cloudletSubmit(Cloudlet, double) - Method in class org.cloudbus.cloudsim.CloudletSchedulerDynamicWorkload
:   Receives an cloudlet to be executed in the VM managed by this scheduler.

cloudletSubmit(Cloudlet, double) - Method in class org.cloudbus.cloudsim.CloudletSchedulerSpaceShared
:   Receives an cloudlet to be executed in the VM managed by this scheduler.

cloudletSubmit(Cloudlet) - Method in class org.cloudbus.cloudsim.CloudletSchedulerSpaceShared


cloudletSubmit(Cloudlet, double) - Method in class org.cloudbus.cloudsim.CloudletSchedulerTimeShared
:   Receives an cloudlet to be executed in the VM managed by this scheduler.

cloudletSubmit(Cloudlet) - Method in class org.cloudbus.cloudsim.CloudletSchedulerTimeShared


cloudletSubmit(Cloudlet, double) - Method in class org.cloudbus.cloudsim.network.datacenter.NetworkCloudletSpaceSharedScheduler
:   Receives an cloudlet to be executed in the VM managed by this scheduler.

cloudletSubmit(Cloudlet) - Method in class org.cloudbus.cloudsim.network.datacenter.NetworkCloudletSpaceSharedScheduler


cloudletSubmittedList - Variable in class org.cloudbus.cloudsim.DatacenterBroker
:   The cloudlet submitted list.

cloudletWaitingList(List<T>) - Method in class org.cloudbus.cloudsim.CloudletSchedulerSpaceShared
:   Cloudlet waiting list.

cloudletWaitingList(List<T>) - Method in class org.cloudbus.cloudsim.network.datacenter.NetworkCloudletSpaceSharedScheduler
:   Cloudlet waiting list.

CloudSim - Class in org.cloudbus.cloudsim.core
:   This class extends the CloudSimCore to enable network simulation in CloudSim.

CloudSim() - Constructor for class org.cloudbus.cloudsim.core.CloudSim


CloudSimExample1 - Class in org.cloudbus.cloudsim.examples
:   A simple example showing how to create a datacenter with one host and run one
    cloudlet on it.

CloudSimExample1() - Constructor for class org.cloudbus.cloudsim.examples.CloudSimExample1


CloudSimExample2 - Class in org.cloudbus.cloudsim.examples
:   A simple example showing how to create
    a datacenter with one host and run two
    cloudlets on it.

CloudSimExample2() - Constructor for class org.cloudbus.cloudsim.examples.CloudSimExample2


CloudSimExample3 - Class in org.cloudbus.cloudsim.examples
:   A simple example showing how to create
    a datacenter with two hosts and run two
    cloudlets on it.

CloudSimExample3() - Constructor for class org.cloudbus.cloudsim.examples.CloudSimExample3


CloudSimExample4 - Class in org.cloudbus.cloudsim.examples
:   A simple example showing how to create
    two datacenters with one host each and
    run two cloudlets on them.

CloudSimExample4() - Constructor for class org.cloudbus.cloudsim.examples.CloudSimExample4


CloudSimExample5 - Class in org.cloudbus.cloudsim.examples
:   A simple example showing how to create
    two datacenters with one host each and
    run cloudlets of two users on them.

CloudSimExample5() - Constructor for class org.cloudbus.cloudsim.examples.CloudSimExample5


CloudSimExample6 - Class in org.cloudbus.cloudsim.examples
:   An example showing how to create
    scalable simulations.

CloudSimExample6() - Constructor for class org.cloudbus.cloudsim.examples.CloudSimExample6


CloudSimExample7 - Class in org.cloudbus.cloudsim.examples
:   An example showing how to pause and resume the simulation,
    and create simulation entities (a DatacenterBroker in this example)
    dynamically.

CloudSimExample7() - Constructor for class org.cloudbus.cloudsim.examples.CloudSimExample7


CloudSimExample8 - Class in org.cloudbus.cloudsim.examples
:   An example showing how to create simulation entities
    (a DatacenterBroker in this example) in run-time using
    a globar manager entity (GlobalBroker).

CloudSimExample8() - Constructor for class org.cloudbus.cloudsim.examples.CloudSimExample8


CloudSimExample8.GlobalBroker - Class in org.cloudbus.cloudsim.examples


CloudSimExample8.GlobalBroker(String) - Constructor for class org.cloudbus.cloudsim.examples.CloudSimExample8.GlobalBroker


CloudSimShutdown - Class in org.cloudbus.cloudsim.core
:   CloudimShutdown waits for termination of all CloudSim user entities to determine the end of
    simulation.

CloudSimShutdown(String, int) - Constructor for class org.cloudbus.cloudsim.core.CloudSimShutdown
:   Allocates a new CloudSimShutdown object.

CloudSimTags - Class in org.cloudbus.cloudsim.core
:   Contains various static command tags that indicate a type of action that needs to be undertaken
    by CloudSim entities when they receive or send events.

ClusteringEngine - Class in org.workflowsim
:   ClusteringEngine is an optional component of WorkflowSim and it merges tasks
    into jobs

ClusteringEngine(String, int) - Constructor for class org.workflowsim.ClusteringEngine
:   Created a new ClusteringEngine object.

ClusteringInterface - Interface in org.workflowsim.clustering
:   The ClusteringInterface for all clustering methods

ClusteringParameters - Class in org.workflowsim.utils
:   ClusteringParameters contains all the parameters used in task clustering

ClusteringParameters(int, int, ClusteringParameters.ClusteringMethod, String) - Constructor for class org.workflowsim.utils.ClusteringParameters
:   Initialize a ClusteringParameters

ClusteringParameters.ClusteringMethod - Enum in org.workflowsim.utils
:   Supported Clustering Method, by default it is none

ClusterStorage - Class in org.workflowsim
:   ClusterStorage is an extention of HarddriveStorage and it is used as a local
    storage system of a vm

ClusterStorage(String, double) - Constructor for class org.workflowsim.ClusterStorage
:   Initialize a ClusterStorage

COMMUNICATION\_LENGTH - Static variable in class org.cloudbus.cloudsim.network.datacenter.NetworkConstants


compareTo(SimEvent) - Method in class org.cloudbus.cloudsim.core.SimEvent


compareTo(Object) - Method in class org.cloudbus.cloudsim.network.datacenter.NetworkCloudlet


compareTo(Object) - Method in class org.cloudbus.cloudsim.network.datacenter.NetworkVm


CondorVM - Class in org.workflowsim
:   Condor Vm extends a VM: the difference is it has a locl storage system and it
    has a state to indicate whether it is busy or not

CondorVM(int, int, double, int, int, long, long, String, CloudletScheduler) - Constructor for class org.workflowsim.CondorVM
:   Creates a new CondorVM object.

Constants - Class in org.cloudbus.cloudsim.examples.power
:   If you are using any algorithms, policies or workload included in the power package, please cite
    the following paper:
    Anton Beloglazov, and Rajkumar Buyya, "Optimal Online Deterministic Algorithms and Adaptive
    Heuristics for Energy and Performance Efficient Dynamic Consolidation of Virtual Machines in
    Cloud Data Centers", Concurrency and Computation: Practice and Experience (CCPE), Volume 24,
    Issue 13, Pages: 1397-1420, John Wiley & Sons, Ltd, New York, USA, 2012

Constants() - Constructor for class org.cloudbus.cloudsim.examples.power.Constants


Consts - Class in org.cloudbus.cloudsim
:   Defines common constants, used throughout cloudsim.

contains(File) - Method in class org.cloudbus.cloudsim.Datacenter
:   Checks whether the resource has the given file.

contains(String) - Method in class org.cloudbus.cloudsim.Datacenter
:   Checks whether the resource has the given file.

contains(String) - Method in class org.cloudbus.cloudsim.HarddriveStorage
:   Checks whether a certain file is on the storage or not.

contains(File) - Method in class org.cloudbus.cloudsim.HarddriveStorage
:   Checks whether a certain file is on the storage or not.

contains(String) - Method in interface org.cloudbus.cloudsim.Storage
:   Checks whether a file is stored in the storage or not.

contains(File) - Method in interface org.cloudbus.cloudsim.Storage
:   Checks whether a file is stored in the storage or not.

containsFile(String) - Static method in class org.workflowsim.utils.ReplicaCatalog
:   Checks whether a file exists

ContinuousDistribution - Interface in org.cloudbus.cloudsim.distributions
:   Interface to be implemented by a random number generator.

copyValue(FileAttribute) - Method in class org.cloudbus.cloudsim.FileAttribute
:   Copy the values of this object into another FileAttribute class

costPerBw - Variable in class org.cloudbus.cloudsim.Cloudlet
:   The cost per bw.

countNonZeroBeginning(double[]) - Static method in class org.cloudbus.cloudsim.util.MathUtil
:   Count non zero beginning of the data.

countShortestRow(double[][]) - Static method in class org.cloudbus.cloudsim.util.MathUtil
:   Count shortest row.

CPUfinTimeCPU - Variable in class org.cloudbus.cloudsim.network.datacenter.NetworkHost
:   time when last job will finish on CPU1

CREATE - Static variable in class org.cloudbus.cloudsim.core.SimEvent


createBroker() - Static method in class org.cloudbus.cloudsim.examples.power.Helper
:   Creates the broker.

createCloudletList(List<Integer>) - Method in class org.cloudbus.cloudsim.examples.network.datacenter.TestBagofTaskApp


createCloudletList(int, int) - Static method in class org.cloudbus.cloudsim.examples.power.random.RandomHelper
:   Creates the cloudlet list.

createCloudletList(List<Integer>) - Method in class org.cloudbus.cloudsim.network.datacenter.AppCloudlet
:   An example of creating APPcloudlet

createCloudletList(List<Integer>) - Method in class org.cloudbus.cloudsim.network.datacenter.WorkflowApp


createCloudletListPlanetLab(int, String) - Static method in class org.cloudbus.cloudsim.examples.power.planetlab.PlanetLabHelper
:   Creates the cloudlet list planet lab.

CREATED - Static variable in class org.cloudbus.cloudsim.Cloudlet
:   The Cloudlet has been created and added to the CloudletList object.

createDatacenter(String, Class<? extends Datacenter>, List<PowerHost>, VmAllocationPolicy) - Static method in class org.cloudbus.cloudsim.examples.power.Helper
:   Creates the datacenter.

createHostList(int) - Static method in class org.cloudbus.cloudsim.examples.power.Helper
:   Creates the host list.

createLinearRegression(double[], double[]) - Static method in class org.cloudbus.cloudsim.util.MathUtil


createLinearRegression(double[][], double[]) - Static method in class org.cloudbus.cloudsim.util.MathUtil


createvmflag - Variable in class org.cloudbus.cloudsim.network.datacenter.NetDatacenterBroker


createVmList(int, int) - Static method in class org.cloudbus.cloudsim.examples.power.Helper
:   Creates the vm list.

createVmsInDatacenter(int) - Method in class org.cloudbus.cloudsim.DatacenterBroker
:   Create the virtual machines in a datacenter.

createVmsInDatacenterBase(int) - Method in class org.cloudbus.cloudsim.network.datacenter.NetDatacenterBroker
:   Create the virtual machines in a datacenter and submit/schedule cloudlets to them.

createWeigthedLinearRegression(double[], double[], double[]) - Static method in class org.cloudbus.cloudsim.util.MathUtil


CTLG\_ADD\_MASTER - Static variable in class org.cloudbus.cloudsim.DataCloudTags
:   Denotes the request to register / add a master file to the Replica Catalogue.  
    The format of this request is Object[3] = {String filename, FileAttribute attr, Integer
    resID}.  
    The reply tag name is `DataCloudTags.CTLG_ADD_MASTER_RESULT`.

CTLG\_ADD\_MASTER\_ERROR - Static variable in class org.cloudbus.cloudsim.DataCloudTags
:   Denotes that master file addition is failed due to an unknown error

CTLG\_ADD\_MASTER\_ERROR\_FULL - Static variable in class org.cloudbus.cloudsim.DataCloudTags
:   Denotes that master file addition is failed due to the catalogue is full

CTLG\_ADD\_MASTER\_RESULT - Static variable in class org.cloudbus.cloudsim.DataCloudTags
:   Sends the result of registering a master file back to sender.  
    The format of the reply is Object[3] = {String filename, Integer uniqueID, Integer resultID}.  
    NOTE: The result id is in the form of CTLG\_ADD\_MASTER\_XXXX where XXXX means the error/success
    message

CTLG\_ADD\_MASTER\_SUCCESSFUL - Static variable in class org.cloudbus.cloudsim.DataCloudTags
:   Denotes that master file addition is successful

CTLG\_ADD\_REPLICA - Static variable in class org.cloudbus.cloudsim.DataCloudTags
:   Denotes the request to register / add a replica file to the Replica Catalogue.  
    The format of this request is Object[2] = {String lfn, Integer resourceID}.  
    The reply tag name is `DataCloudTags.CTLG_ADD_REPLICA_RESULT`.

CTLG\_ADD\_REPLICA\_ERROR - Static variable in class org.cloudbus.cloudsim.DataCloudTags
:   Denotes that replica file addition is failed due to an unknown error

CTLG\_ADD\_REPLICA\_ERROR\_DOESNT\_EXIST - Static variable in class org.cloudbus.cloudsim.DataCloudTags
:   Denotes that replica file addition is failed because the given file name does not exist in
    the catalogue

CTLG\_ADD\_REPLICA\_ERROR\_FULL - Static variable in class org.cloudbus.cloudsim.DataCloudTags
:   Denotes that replica file addition is failed due to the catalogue is full

CTLG\_ADD\_REPLICA\_RESULT - Static variable in class org.cloudbus.cloudsim.DataCloudTags
:   Sends the result of registering a replica file back to sender.  
    The format of the reply is Object[2] = {String lfn, Integer resultID}.  
    NOTE: The result id is in the form of CTLG\_ADD\_REPLICA\_XXXX where XXXX means the
    error/success message

CTLG\_ADD\_REPLICA\_SUCCESSFUL - Static variable in class org.cloudbus.cloudsim.DataCloudTags
:   Denotes that replica file addition is successful

CTLG\_DELETE\_MASTER - Static variable in class org.cloudbus.cloudsim.DataCloudTags
:   Denotes the request to de-register / delete a master file from the Replica Catalogue.  
    The format of this request is Object[2] = {String lfn, Integer resourceID}.  
    The reply tag name is `DataCloudTags.CTLG_DELETE_MASTER_RESULT`.

CTLG\_DELETE\_MASTER\_DOESNT\_EXIST - Static variable in class org.cloudbus.cloudsim.DataCloudTags
:   Denotes that master file deletion is failed because the file does not exist in the catalogue

CTLG\_DELETE\_MASTER\_ERROR - Static variable in class org.cloudbus.cloudsim.DataCloudTags
:   Denotes that master file deletion is failed due to an unknown error

CTLG\_DELETE\_MASTER\_REPLICAS\_EXIST - Static variable in class org.cloudbus.cloudsim.DataCloudTags
:   Denotes that master file deletion is failed because replica files are still in the catalogue.

CTLG\_DELETE\_MASTER\_RESULT - Static variable in class org.cloudbus.cloudsim.DataCloudTags
:   Sends the result of de-registering a master file back to sender.  
    The format of the reply is Object[2] = {String lfn, Integer resultID}.  
    NOTE: The result id is in the form of CTLG\_DELETE\_MASTER\_XXXX where XXXX means the
    error/success message

CTLG\_DELETE\_MASTER\_SUCCESSFUL - Static variable in class org.cloudbus.cloudsim.DataCloudTags
:   Denotes that master file deletion is successful

CTLG\_DELETE\_REPLICA - Static variable in class org.cloudbus.cloudsim.DataCloudTags
:   Denotes the request to de-register / delete a replica file from the Replica Catalogue.  
    The format of this request is Object[2] = {String lfn, Integer resourceID}.  
    The reply tag name is `DataCloudTags.CTLG_DELETE_REPLICA_RESULT`.

CTLG\_DELETE\_REPLICA\_ERROR - Static variable in class org.cloudbus.cloudsim.DataCloudTags
:   Denotes that replica file deletion is failed due to an unknown error

CTLG\_DELETE\_REPLICA\_ERROR\_DOESNT\_EXIST - Static variable in class org.cloudbus.cloudsim.DataCloudTags
:   Denotes that replica file deletion is failed because the file does not exist in the catalogue

CTLG\_DELETE\_REPLICA\_RESULT - Static variable in class org.cloudbus.cloudsim.DataCloudTags
:   Sends the result of de-registering a replica file back to sender.  
    The format of the reply is Object[2] = {String lfn, Integer resultID}.  
    NOTE: The result id is in the form of CTLG\_DELETE\_REPLICA\_XXXX where XXXX means the
    error/success message

CTLG\_DELETE\_REPLICA\_SUCCESSFUL - Static variable in class org.cloudbus.cloudsim.DataCloudTags
:   Denotes that replica file deletion is successful

CTLG\_FILE\_ATTR\_DELIVERY - Static variable in class org.cloudbus.cloudsim.DataCloudTags
:   Sends the result for a file attribute back to sender.  
    The format of the reply is {FileAttribute fileAttr}.  
    NOTE: The fileAttr could be null if not found.

CTLG\_FILTER - Static variable in class org.cloudbus.cloudsim.DataCloudTags
:   Denotes the request to get a list of file attributes based on the given filter.  
    The format of this request is Object[2] = {Filter filter, Integer senderID}.  
    The reply tag name is `DataCloudTags.CTLG_FILTER_DELIVERY`.

CTLG\_FILTER\_DELIVERY - Static variable in class org.cloudbus.cloudsim.DataCloudTags
:   Sends the result for a list of file attributes back to sender.  
    The format of the reply is {List attrList}.  
    NOTE: The attrList could be null if not found.

CTLG\_GET\_FILE\_ATTR - Static variable in class org.cloudbus.cloudsim.DataCloudTags
:   Denotes the request to get the attribute of a file.  
    The format of this request is Object[2] = {String lfn, Integer senderID}.  
    The reply tag name is `DataCloudTags.CTLG_FILE_ATTR_DELIVERY`.

CTLG\_GET\_REPLICA - Static variable in class org.cloudbus.cloudsim.DataCloudTags
:   Denotes the request for a location of a replica file.  
    The format of this request is Object[2] = {String lfn, Integer senderID}.  
    The reply tag name is `DataCloudTags.CTLG_REPLICA_DELIVERY`.  
    NOTE: This request only ask for one location only not all.

CTLG\_GET\_REPLICA\_LIST - Static variable in class org.cloudbus.cloudsim.DataCloudTags
:   Denotes the request for all locations of a replica file.  
    The format of this request is Object[2] = {String lfn, Integer senderID}.  
    The reply tag name is `DataCloudTags.CTLG_REPLICA_LIST_DELIVERY`.

CTLG\_MODIFY\_MASTER - Static variable in class org.cloudbus.cloudsim.DataCloudTags
:   Denotes the request to modify an existing master file information stored in the Replica
    Catalogue.  
    The format of this request is Object[3] = {String filename, FileAttribute attr, Integer
    resID}.  
    The reply tag name is `DataCloudTags.CTLG_MODIFY_MASTER_RESULT`.

CTLG\_MODIFY\_MASTER\_ERROR - Static variable in class org.cloudbus.cloudsim.DataCloudTags
:   Denotes that master file modification is failed due to an unknown error

CTLG\_MODIFY\_MASTER\_ERROR\_DOESNT\_EXIST - Static variable in class org.cloudbus.cloudsim.DataCloudTags
:   Denotes that master file modification is failed because the file does not exist in the
    catalogue

CTLG\_MODIFY\_MASTER\_ERROR\_READ\_ONLY - Static variable in class org.cloudbus.cloudsim.DataCloudTags
:   Denotes that master file modification is failed because the file attribute is set to a
    read-only

CTLG\_MODIFY\_MASTER\_RESULT - Static variable in class org.cloudbus.cloudsim.DataCloudTags
:   Sends the result of modifying a master file back to sender.  
    The format of the reply is Object[2] = {String lfn, Integer resultID}.  
    NOTE: The result id is in the form of CTLG\_MODIFY\_MASTER\_XXXX where XXXX means the
    error/success message

CTLG\_MODIFY\_MASTER\_SUCCESSFUL - Static variable in class org.cloudbus.cloudsim.DataCloudTags
:   Denotes that master file deletion is successful

CTLG\_REPLICA\_DELIVERY - Static variable in class org.cloudbus.cloudsim.DataCloudTags
:   Sends the result for a location of a replica file back to sender.  
    The format of the reply is Object[2] = {String lfn, Integer resourceID}.  
    NOTE: The resourceID could be -1 if not found.

CTLG\_REPLICA\_LIST\_DELIVERY - Static variable in class org.cloudbus.cloudsim.DataCloudTags
:   Sends the result for all locations of a replica file back to sender.  
    The format of the reply is Object[2] = {String lfn, List locationList}.  
    NOTE: The locationList could be null if not found.

currentAppId - Static variable in class org.cloudbus.cloudsim.network.datacenter.NetworkConstants


currentCloudletId - Static variable in class org.cloudbus.cloudsim.network.datacenter.NetworkConstants


currentCpus - Variable in class org.cloudbus.cloudsim.CloudletSchedulerSpaceShared
:   The current CPUs.

currentCPUs - Variable in class org.cloudbus.cloudsim.CloudletSchedulerTimeShared
:   The current cp us.

currentCpus - Variable in class org.cloudbus.cloudsim.network.datacenter.NetworkCloudletSpaceSharedScheduler
:   The current CPUs.

currStagenum - Variable in class org.cloudbus.cloudsim.network.datacenter.NetworkCloudlet

A B C D E F G H I J L M N O P Q R S T U V W Z

- Overview
- Package
- Class
- Use
- Tree
- Deprecated
- Index
- Help

- Prev Letter
- Next Letter

- Frames
- No Frames

- All Classes
